# MiR-155, a potential serum marker of extramammary Paget’s disease

**DOI:** 10.1186/s12885-018-4994-1

**Published:** 2018-11-07

**Authors:** Hao Guo, Rui-Qun Qi, Jie Sheng, Chang Liu, Hang Ma, He-Xiao Wang, Jiu-Hong Li, Xing-Hua Gao, Yin-Sheng Wan, Hong-Duo Chen

**Affiliations:** 1 Department of Dermatology, No.1 Hospital of China Medical University, 155N. Nanjing Street, Shenyang, 110001 People’s Republic of China; 2 Department of Anesthesiology, No.1 Hospital of China Medical University, 155N. Nanjing Street, Shenyang, 110001 People’s Republic of China; 3 Department of Biomedical and Pharmaceutical Sciences, College of Pharmacy, University of Rhode Island, Kingston, RI 02881 USA; 4 Department of Physiology, Providence College, Providence, RI 02918 USA

**Keywords:** General dermatology, MiR-155, Extramammary Paget’s disease

## Abstract

**Background:**

Extramammary Paget’s disease (EMPD), a rare skin malignancy with non-specific manifestations, is often misdiagnosed as eczema of scrotum or tinea cruris. Although the diagnosis of EMPD could be confirmed by biopsy, it can be delayed as patients are reluctant to receive invasive operations. Herein, we investigated the serum miRNA expressions of EMPD patients and compared to that of the eczema of scrotum or tinea cruris patients as well as health volunteers for potential diagnostic markers for EMPD.

**Methods:**

Altogether 45 subjects including 16 patients diagnosed with EMPD, 12 patients diagnosed with eczema of scrotum or tinea cruris and 17 healthy volunteers were enrolled in this study. Serum from all of subjects were collected to identify miRNAs (by miRNA array global normalization, RT-PCR validation, and receiver operating characteristic curve analysis) that could be potential diagnostic markers for EMPD.

**Results:**

The miRNA array analyses revealed that the expressions of 37 miRNAs from the EMPD patients were different (change ≥4-fold) from health volunteers. Among these miRNAs, the expression of miR-155 was significantly increased (*p* < 0.01) in the EMPD patients as compared with that of the health volunteers and the eczema of scrotum or the tinea cruris patients (no difference between these two control groups). In addition, receiver operating characteristic (ROC) curve analysis showed that diagnostic capacities (defined as the area under curve of ROC) of miR-155 are 0.85 (as compared with health volunteers group) and 0.81 (as compared with the eczema of scrotum or the tinea cruris patients group), respectively.

**Conclusion:**

The serum miRNA expression of gene miR-155 in the EMPD patients was differentiated from that of other subjects warranting further validation of miR-155 as a diagnostic marker of EMPD.

## Introduction

Extramammary Paget’s disease (EMPD) is a rare cutaneous malignancy that affects apocrine-rich areas such as the vulva, penis, scrotum and perianal area [1]. Its clinical manifestations are not specific and can be presented as erythematous patches, plaques or erosions, occasionally with adherent crust [2]. Patients may also experience symptoms of pruritus and tenderness [1–3]. Therefore, EMPD is also known as “eczematoid carcinoma” as it can be misdiagnosed as eczema of scrotum (ECZ) or tinea cruris (TIN) at the early stage. In the clinic, without proper histopathological examinations, EMPD patients with unspecific manifestations are often misdiagnosed as ECZ/TIN. Topical treatments may relieve symptoms of ECZ/TIN (such as pruritus), it gives an illusion of false improvement which leads to further delayed diagnosis of EMPD. It is reported that EMPD has a median delayed diagnosis of 2 years due to the aforementioned reasons [2, 4]. Although biopsy is the gold standard for diagnosis of EMPD, some suspected EMPD patients are reluctant to receive biopsy due to the invasiveness of the procedure. Therefore, investigations of noninvasive markers to improve the diagnosis of EMPD are of great research interest.

MicroRNAs (miRNAs) are a class of 19–25-nucleotide noncoding RNAs that have been implicated in the regulations of various cellular processes [5]. Some miRNAs have been reported to have significant correlations with cancer development and progression. They have been investigated as circular markers of many malignancies [6, 7]. In our previous study, miRNAs including miR-31, miR-375 and miR-31* were overexpressed in EMPD tissue as compared to normal keratinocytes and normal apocrine glands (the historical origin of EMPD) using laser-capture microdissection in frozen tissues from 12 EMPD patients [8]. However, to date, neither serum miRNAs of EMPD have been investigated, nor serum markers were validated in clinics for the diagnosis of EMPD. Herein, we investigated expressions of miRNAs from human subjects using miRNA array analyses to identify specific miRNAs for potential diagnostic serum markers of EMPD.

## Patients and methods

### Study population

The study was approved by ethics committee of No. 1 Hospital of China Medical University (No. AF-SOP-07-1.0-01).

Patients and healthy volunteers were enrolled from No.1 Hospital of China Medical University from 2014 to 2017. Healthy volunteers (17), EMPD patients (16), ECZ/TIN patients (12) were archived and enrolled in this study. Clinical basic characteristics of the volunteers and patients were summarized in Table 1. To minimize the heterogeneity in our enrolled cases, females were excluded in this study. All of the patients and healthy volunteers came from the region of northeastern of China.

**Table 1:** Demographics and clinical features

Characteristics	Normal	EMPD	ECZ/ TIN
Sample size, *n*	17	16	12 (n_TIN_ = 7; n_ECZ_ = 5)
Gender, *n*			
Male	17	16	12
Female	0	0	0
Age, years	61.8 ± 8.7	63.7 ± 8.4	58.2 ± 11.7
Primary site, *n*			
Penis		1	0
Scortum		5	5
Scortum & penis		1	0
Gorin (including thigh)		5	4
Scortum & gorin		4	3
Size of lesion (cm^2^), *n*			
< 10		2	1
10–19		4	4
20–29		6	4
29–39		3	3
> 39		1	0

Enrolled healthy volunteers (normal group) were excluded from any malignancies, allergic dermatisis, infections and certain internal diseases through physical examinations as normal control.

EMPD patients (EMPD group), diagnosed via histopathological examination, were ruled out of diseases including allergic dermatisis, infections, EMPD metastasis and other malignancies via comprehensive examinations (including chest CT, abdominal & urinary system B ultrasonic and enteroscope).

Eczema of scrotum or tinea cruris patients (ECZ/TIN group) were diagnosed via comprehensive physical and dermatological examinations (including blood routine, total IgE and fungus microscope examination). They were ruled out of other infections and malignancies via comprehensive examinations.

### RNA isolation

Blood samples were collected form enrolled patients and volunteers. Serum from blood samples were prepared through a centrifuge at 4000 rpm for 15 min. Collected serum samples were extracted for total RNA including miRNAs by Qiagen miRNeasy Micro Kit accordingly to the manufacture’s protocol with minor modifications. Briefly, serum from each sample was lysed with QIAzol lysis reagent and then 100 uL of chloroform were added. Next, samples were centrifuged for 15 min at 12000 xg at 4 °C. The upper aqueous phase of each sample was transferred and 1.5 times of volumes of 100% ethanol were added. Then mixtures went through the RNeasy Micro spin column and 40 ul RNase free water were used to elute. The volume of each sample was reduced to 15–20 ul by vacuo. RNA quantity (15–20 ng/ul) and quality (OD 260/280 was approximately 1.0 with a peak at 270 nm) were assessed with the ThermoScientific NanoDrop 2000 (Thermo Fisher Scientific, Inc., Franklin, MA, USA).

### TaqMan low-density array miRNA qRT-PCR

Due to the limited RNA obtained from serum, a pre-amplification step was added as per manufacture’s protocol when miRNA array was performed in 2 samples of normal and 2 samples of EMPD in both groups for screening.

The reversed transcription of RNA was prepared using the TaqMan MiRNA Reverse Transcription Kit and TaqMan MiRNA Multiplex RT Assays (human pool A; V2.1). RNA (3 ul) was added to each reaction and RT-PCR was carried on an ABI Veriti Thermal cycler (Applied Biosystems, Foster City, CA, USA). The product (2.5 ul) from each reaction was pre-amplified as per manufacturer’s protocol with the Megaplex PreAmp Primers (10×), Human pool A (V2.1) and TaqMan PreAmp Master Mix (2×). Then miRNA expression was profiled with TaqMan Human MicroRNA array card A (V2.1), performed on a 7900HT Fast Real-Time PCR System (Applied Biosystems, Foster City, CA, USA), using the manufacturer’s recommended protocol.

### Global normalization

Raw cycle threshold (Ct) values were calculated using SDS 2.3 and RQ manager 1.2 software (Applied Biosystems*,* Foster City, CA, USA) using default baselines and threshold settings. All the Ct values were exported into StatMiner® 4.2 (Integromics® Inc., Philadelphia, PA, USA) for global normalization. The miRNAs detected in all of the samples were used for global normalization. The mean Ct value of each individual sample was calculated by the global normalization, and subtracts the Ct value from each miRNA of the same sample to obtain the ∆Ct value. The miRNAs that were expressed in all samples were considered meaningful for further data analysis. Heat-map analysis was performed using ∆Ct with hierarchical clustering using miRNAs expressed in all 4 samples. Average ∆Ct of normal and EMPD were calculated separately, the ∆∆Ct (∆Ct_NORMAL_-∆Ct_EMPD_) was also calculated for further study.

### RT-PCR

After significantly different expressed miRNAs were obtained, specific RT-PCR analyses were performed (15 normal, 14 EMPD as well as 12 ECZ/TIN). The RNA was reverse transcribed using the TaqMan MiRNA Reverse Transcription Kit. Then RT- PCR was performed following protocols provided by TaqMan.

### Statistical analysis

For all samples in RT-PCR, cycle threshold (Ct) values were obtained with SDS 2.3 and RQ manager 1.2 software (Applied Biosystems, Foster City, CA, USA). Expressions of miRNAs were calculated with StatMiner® 4.2 (Integromics® Inc., Philadelphia, PA, USA) using –ΔCt* [−(Ct-Ct_mir-374-5p_)] of each sample. Bonferroni’s Multiple Comparison Test was used to compare the (-ΔCt*) value among the three groups. *P*-value < 0.05 are considered significantly different between two groups. Receiver Operating Characteristic (ROC) curve analysis was then performed using the differentially expressed miRNA. Scattergraph and ROC curve analysis were both performed by GraphPad Prism.

## Results

### MiRNA array and global normalization

**Fig. 1:**
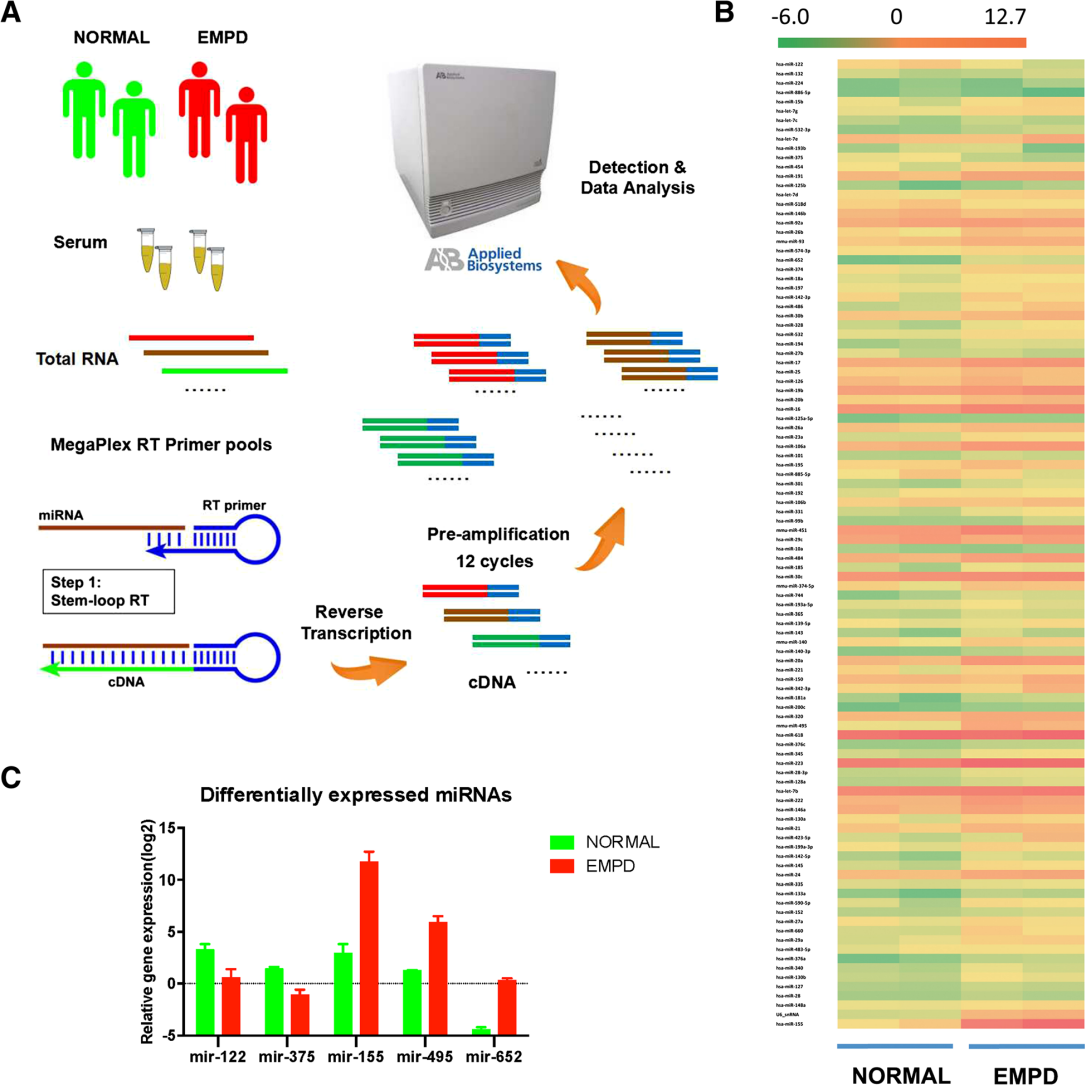
EMPD’s procedure chart and result of miRNA array. **a** Flow diagram of how our serum miRNA array was performed. **b** MiRNA expression profiles. MiRNA expressions of two pairs of normal and healthy volunteers and EMPD patients were profiled using TaqMan Human MicroRNA array card A (v2.1). The cycle threshold (Ct) values were obtained with SDS 2.3 and RQ manager 1.2 software (Applied Biosystem) and analyzed with RealTime StatMiner® 4.2 software (Integromics, Inc.). The -ΔCt was calculated and heat map analysis was performed with Excel. A green-yellow-red color scale (− 6.0 to 12.7) depicts normalized miRNAs expression level based on global normalization. **c** Differently expressed miRNAs between normal and EMPD, using -ΔCt from 2 decreased miRNAs (miR-122, miR-375) and 3 most significantly increased miRNAs (miR-155, miR-495, miR-652)

The miRNA gene expression profiles were obtained from two normal and two EMPD patients (flow diagram showed how serum miRNA arrays analyses shown in Fig. 1a). A total of 255 miRNAs were detected in the TaqMan® Array MicroRNA human card A. In average 166 miRNAs were detected per sample, and 105 miRNAs were detected in all the samples. For each sample, the global mean value of the expression of the 105 miRNAs was calculated, then the difference (∆Ct) between the expression of each individual miRNA in this given sample and the global mean was obtained. Heat-map analysis showed that most of miRNAs’ expressions were relatively consistent across different samples. The heat-map indicated that global normalization reduced the variations, and the overall miRNA gene expression profiles between different individuals were consistent (Fig. 1b). Some miRNAs showed difference between normal and EMPD groups [miRNAs (|ΔΔCt| ≥ 2)] and were defined as potentially different. Expression of 2 miRNAs were down-regulated and 35 miRNAs were up-regulated (Table 2).

**Table 2:** Differentially expressed miRNAs in array

miRNAs	ΔCt_Normal-1_	ΔCt_Normal-2_	ΔCt _EMPD-1_	ΔCt _EMPD-2_	ΔΔCt
hsa-miR-375	−1.2	− 1.6	0.6	1.6	−2.5
hsa-miR-122	−2.7	−3.8	−1.4	0.3	−2.7
hsa-miR-155	−2.0	−3.8	−10.7	−12.7	8.8
hsa-miR-652	4.7	4.2	−0.5	−0.1	4.8
mmu-miR-495	−1.3	−1.2	−6.5	−5.3	4.7
hsa-miR-486	0.8	0.1	−2.2	−4.1	3.6
hsa-miR-376a	4.7	3.0	0.8	0.4	3.3
hsa-miR-423-5p	−0.4	0.8	−0.5	−5.2	3.11
hsa-miR-133a	3.0	4.9	0.7	1.2	3.0
hsa-miR-20a	−5.2	−4.6	−8.2	−7.7	3.0
hsa-miR-181a	1.7	4.6	0.6	0.1	2.79
hsa-miR-744	3.2	1.5	−0.7	−0.1	2.75
hsa-miR-660	−0.3	0.0	−3.5	−2.1	2.63
hsa-miR-29a	−0.1	−1.2	−3.1	−3.4	2.6
hsa-miR-142-5p	1.4	3.0	−0.8	0.0	2.6
hsa-miR-145	−0.1	0.9	−2.4	−1.9	2.6
hsa-miR-590-5p	−0.7	1.6	−2.3	−1.7	2.4
hsa-miR-106a	−5.4	−5.9	−8.0	−8.0	2.4
hsa-miR-328	0.3	1.5	−1.2	−1.7	2.4
hsa-miR-140-3p	3.2	3.0	1.1	0.4	2.35
hsa-miR-194	2.2	1.3	−0.8	−0.4	2.4
hsa-miR-532-3p	2.9	2.4	0.9	0.0	2.2
hsa-miR-23a	−0.1	0.0	−2.4	−2.1	2.2
hsa-miR-223	−10.6	−10.1	−12.8	−12.3	2.2
hsa-miR-26b	−2.6	−1.5	−4.6	−3.8	2.2
hsa-miR-17	−6.0	−6.0	−8.1	−8.3	2.2
hsa-miR-185	0.0	1.9	−1.3	−1.0	2.1
hsa-miR-20b	−3.2	−2.6	−4.8	−5.2	2.1
hsa-miR-454	−1.6	0.0	−2.6	−3.1	2.1
hsa-miR-143	1.4	3.4	−0.6	1.2	2.1
hsa-miR-191	−5.2	−4.2	−6.7	−6.7	2.0
hsa-miR-15b	−1.4	0.3	−2.0	−3.1	2.0
hsa-miR-130b	0.4	0.9	−1.7	−1.0	2.0
hsa-miR-200c	4.6	3.9	2.6	2.0	2.0
hsa-miR-301	1.0	1.9	−0.1	−1.0	2.0
hsa-miR-222	−5.5	−5.3	−7.8	−6.9	2.0
hsa-miR-484	−5.7	−5.1	−7.0	−7.7	2.0

### MiR-155 was found obviously up-regulated in EMPD serum

The 2 down-regulated miRNAs (miR-122 and miR-375) and 3 up-regulated miRNAs with largest |ΔΔCt| (miR-155, miR-495, and miR-652) were selected for further validation (Fig. 1c).

**Fig. 2:**
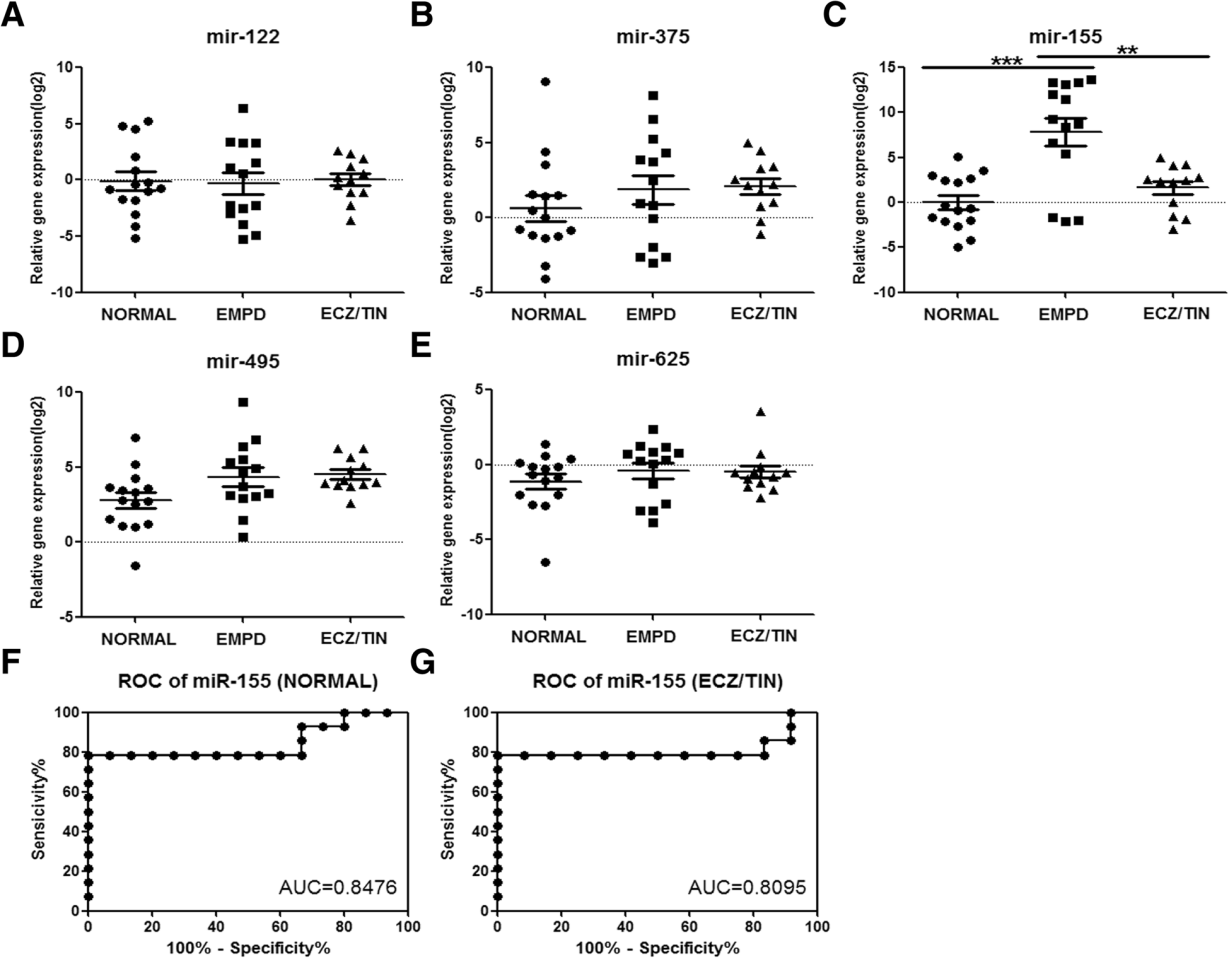
EMPD’s expression of specific miRNAs and ROC curve. **a-e** The expression levels (-ΔCt) of miR-122, miR-375, miR-155, miR-495 and miR-625 in normal, EMPD and ECZ/TIN, using –ΔCt’ [−(Ct-Ctmir-374-5p)] among normal (*n* = 15), EMPD (*n* = 14) and ECZ/TIN groups (*n* = 12) (**p < 0.01, ***p < 0.0001). ROC curve of serum miR-155 in EMPD patients. **f** using normal as control; **g** using ECZ/TIN as control

Then RT-PCR of the above 5 miRNAs among normal, EMPD as well as ECZ/TIN groups (the differential diagnosis group) were performed. Bonferroni’s Multiple Comparison Test was carried out among three groups. Expression levels of miR-122, miR-375, miR-495 and miR-652 showed no difference among three groups (Fig. 2). Expression of miR-155 significantly increased in the EMPD group as compared to other groups and there is no difference between normal and ECZ/TIN (Fig. 2c) groups. We combined data from two common differential diagnosis diseases, ECZ/TIN, together as one group due to the limited patient number we enrolled.

### ROC curve analysis of serum miR-155 of EMPD

The expression of miRNA-155 showed significant increase as compared to normal and common differential diagnosis diseases, which suggested that miRNA-155 may be a potential serum marker of EMPD diagnosis. Furthermore, ROC curve analysis of miR-155 was established based on three groups. Our results showed that miR-155 could accurately distinguish patients of EMPD from healthy volunteers with AUC (area under curve) of 0.85 (Fig. 2f). Similarly, this marker could distinguish patients with EMPD from patients with ECZ/TIN with AUC of 0.81 (Fig. 2g).

## Discussion

Paget’s disease is a rare cutaneous intraepithelial malignancy characterized by Paget cells (large cytoplasm and mucin rich adenocarcinoma cells) [9]. It has two subtypes according to the affected anatomic location: mammary Paget’s diseases and EMPD. The mammary Paget disease was first found by James Paget in 1874, it was reported as an intraepithelial breast carcinoma and suggested that the pattern may be found in other parts [10]. EMPD was first described by Crocker in 1888 [10].

EMPD has unspecific manifestations and can be misdiagnosed as ECZ/TIN. It also has a high recurrence rate after surgery treatment due to the subclinical extension and the multifocal feature [2, 11–14]. Therefore, researches have been focusing on investigations of serum markers aiming to improve the diagnosis and prognosis [15, 16]. Circular miRNAs have been extensively investigated as biomarkers helping with the diagnosis during the past decade [17]. For this current study, a major finding was that miR-155 was significantly increased in the serum of EMPD patients. We sought to identify several promising serum markers, and use the combination of serum markers as a diagnostic method. However, the results of miR-122, miR-375, miR-495 and miR-625 showed no difference between normal and EMPD groups which were differentially expressed in array. This might be caused by the heterogeneity among patients and the limited number enrolled in the array analyses. Based on our previous studies, the serum miRNAs varied immensely among patients [18]. This study was performed on small sample size, and all the other published serum markers of EMPD were based on small sample size research due to the rarity of the disease [15, 16]. We suggest the specific expressed miR-155 can be used and/or in combination with other serum markers including CEA and Cytokeratin 19 fragment 21–1 to improve the diagnosis after further validation [15, 16].

In Fig. 2, three EMPD patients showed relative low expression of serum miR-155. However, no specific clinical features in age, tumor site, tumor size, tumor depth, and appendages involvement in pathology were observed in these patients. It is reported that > 93% of mammary Paget’s diseases was associated with underlying ductal breast cancer [19]. In addition, 29% of EMPD patients were associated with internal malignancies including breast cancer, colorectal cancer, prostate cancer, bladder cancer and gastric cancer [20]. Therefore, EMPD is likely associated with internal malignancies with contiguous epithelium [2]. The EMPD in perianal area is more likely associated with underlying colorectal cancer, while EMPD in scrotum or vulvar have a high incidence of prostate cancer [2].

A large body of evidence reveals that EMPD is an adenoma the histogenetic origin from apocrine glands [1, 21–27]. In histological study, mammary glands and apocrine glands have the same apocrine secretion pattern. Some immunohistochemical marker of EMPD are also positive in specific type of breast cancer such as human epidermal growth factor receptor 2 (Her2/erbB2), carcinoembryonic antigen (CEA), cytokeratin 7 (CK7) and gross cystic disease fluid protein 15 (GCDFP15) [1, 23, 28–32]. These markers are also positive in other cancers such as prostate adenocarcinoma (Her2/erbB2, CK7 and GCDFP15) [33–35] and colon cancer (Her2/erbB2 and CEA) [34, 36, 37]. The above evidence suggested that EMPD may have common features with breast cancer, prostate cancer and colon cancer as they are all adenocarcinomas and have a high incidence of co-existence. In our current study, we found the expression of miRNA-155 significantly increased as compared to healthy volunteers and common differential diagnosis diseases. In our previous study, we performed miRNA arrays between EMPD tissue and peripheral normal skin from the fresh frozen tissue of the same patient using laser-capture microdissection [8]. Data from that study showed that miR-155 was overexpressed in the tissue EMPD (AVE_-△Ct EMPD_ = − 19.96) compared to peripheral normal skin (AVE_-△Ct CON_ = − 22.97) in array [8]. The consistent findings suggest the increased serum miR-155 may be derived from the EMPD tumor tissue. MiR-155 is also overexpressed in the tissue and serum of multiple types of cancers including breast, prostate, and colon cancers [38–44]. MiR-155 is now considered as one of the most familiar onco-miRNAs especially in breast cancer. The overexpressed serum miR-155 in breast cancer has been varifed by different research groups [38–40]. Our result provides new insights that these malignancies may share common features. However, further investigations on the linkage between miR-155 and EMPD are warranted.

## Conclusion

In summary, this is the first study on the serum miRNA expression profile of EMPD patients. Biomarker miR-155 was significantly increased in EMPD patients as compared with the two control groups (health volunteers and ECZ or TIN patients; no difference between these two controls). Therefore, miR-155 could be targeted as a potential diagnostic marker to improve the diagnosis of EMPD warranting further validations.

## Abbreviations

AUC: Area under curve
CEA: Carcinoembryonic antigen
CK7: Cytokeratin 7
Ct: Cycle threshold
ECZ: Eczema of scrotum
ECZ/TIN: Eczema of scrotum or tinea cruris
EMPD: Extramammary Paget’s disease
GCDFP15: Gross cystic disease fluid protein 15
Her2/erbB2: Human epidermal growth factor receptor 2
miRNA: MicroRNA
miRNAs: MicroRNAs
ROC: Receiver Operating Characteristic
TIN: Tinea cruris
